# Dementia and risk of visual impairment in Chinese older adults

**DOI:** 10.1038/s41598-022-22785-x

**Published:** 2022-10-27

**Authors:** Charlotte P. C. Kwok, Jessie O. T. Kwok, Rachel W. K. Yan, Kaspar K. W. Lee, Marcus Richards, Wai C. Chan, Helen F. K. Chiu, Ruby S. Y. Lee, Linda C. W. Lam, Allen T. C. Lee

**Affiliations:** 1 Department of Psychiatry, Faculty of Medicine, The Chinese University of Hong Kong, Hong Kong SAR, China; 2 MRC Unit for Lifelong Health and Ageing at UCL, London, UK; 3 Department of Psychiatry, The University of Hong Kong, Hong Kong SAR, China; 4 Elderly Health Service, Department of Health, The Government of Hong Kong SAR, Hong Kong SAR, China

**Keywords:** Eye diseases, Dementia

## Abstract

We had previously identified visual impairment increasing risk of incident dementia. While a bi-directional vision-cognition association has subsequently been proposed, no study has specifically examined the longitudinal association between dementia and incidence of clinically defined visual impairment. In this territory-wide community cohort study of 10,806 visually unimpaired older adults, we examined their visual acuity annually for 6 years and tested if dementia at baseline was independently associated with higher risk of incident visual impairment (LogMAR ≥ 0.50 in the better eye despite best correction, which is equivalent to moderate visual impairment according to the World Health Organization definition). By the end of Year 6, a total of 3151 (29.2%) participants developed visual impairment. However, we did not find baseline dementia associating with higher risk of incident visual impairment, after controlling for baseline visual acuity, cataract, glaucoma, diabetes, hypertension, hypercholesterolemia, heart diseases, stroke, Parkinson’s disease, depression, hearing and physical impairments, physical, intellectual and social activities, diet, smoking, age, sex, educational level, and socioeconomic status. Among different covariables, baseline visual acuity appears to be more important than dementia in contributing to the development of visual impairment. Our present findings highlight the need for re-evaluating whether dementia is indeed a risk factor for visual impairment.

## Introduction

Dementia and visual impairment are common in older populations. Globally, over 50 million adults aged 55 years or older are living with dementia^1^, and over 200 million adults aged 50 or older are experiencing moderate-to-severe visual impairment^2^.

Visual impairment might be a modifiable risk factor for dementia^3–7^. However, misclassification error (i.e., people with visual impairment scoring worse in cognitive tests that require good vision and thus being misidentified as dementia) and reverse causation (i.e., people in preclinical stage of dementia already experiencing visual disturbance or having trouble in following the instructions of visual acuity test owing to subtle cognitive impairment) might account for the observed association of visual impairment increasing the risk of dementia. We had previously conducted a 6-year longitudinal study, with designs to address these methodological limitations^8^. We found that moderate-to-severe (but not mild) visual impairment—as defined by visual acuity of equal to or worse than 6/18 or LogMAR ≥ 0.5 according to the World Health Organization definition^9^—was independently associated with a higher risk of incident dementia. Our data provide additional evidence to support that visual impairment could be a potential risk factor for dementia, and our findings suggest that early identification and correction of poor visual acuity might help slow or prevent clinical onset of dementia in older adults.

Recently, there has been a growing interest and speculation of presence of a reverse association, with dementia potentially increasing the risk of incident visual impairment^10^. Establishing this association is important because among older adults with dementia, those with co-morbid self-reported visual difficulty appear to have significantly poorer mobility and functioning than those without^11^.

Nevertheless, to our knowledge, no study has specifically examined the longitudinal association of dementia with incident clinically defined visual impairment. While the Salisbury Eye Evaluation Study found that a worse Mini-Mental State Examination (MMSE) score was associated with worse visual acuity in a subsequent round of assessment^12^, the MMSE is a cognitive screening test, and the score is not diagnostic of dementia. Also, the baseline MMSE score was found to have a much smaller effect on subsequent change in visual acuity than the other way around. On the other hand, data from the National Health and Aging Trends Study suggested that baseline dementia was associated with a similarly higher risk of self-reported visual difficulty over time to that of the reverse direction^13^, regardless of the year of cohort examined amid statistical concerns of combining different cohorts together in the analysis^14,15^. While self-reported visual difficulty represents a wider construct and encompasses all dimensions of visual problems, people with dementia might have difficulty in accurately reporting problems relating to their vision. Using self-reported poor vision to identify visual impairment in people with dementia is thus susceptible to misclassification error. One way to minimize this bias is to perform simple standardized clinical assessment of visual acuity.

To identify the association between baseline dementia and risk of incident visual impairment, we followed a large community cohort of older Chinese adults who were free of visual impairment at baseline and received annual visual acuity assessment. We hypothesized that dementia was independently associated with a higher risk of incident visual impairment. Our findings might add to the existing literature of dementia having an important role in the development of visual impairment.

## Results

### Baseline characteristics

Of the 10,806 participants, 6556 (60.7%) were female, and 194 (1.8%) had dementia at baseline. Mean age at baseline was 73.6 years (SD = 4.5 years), and mean visual acuity in LogMAR was 0.37 (SD = 0.08). Median follow-up period was 5.0 years (interquartile range 4.0–6.0 years).

### Incidence of visual impairment

**Figure 1:**
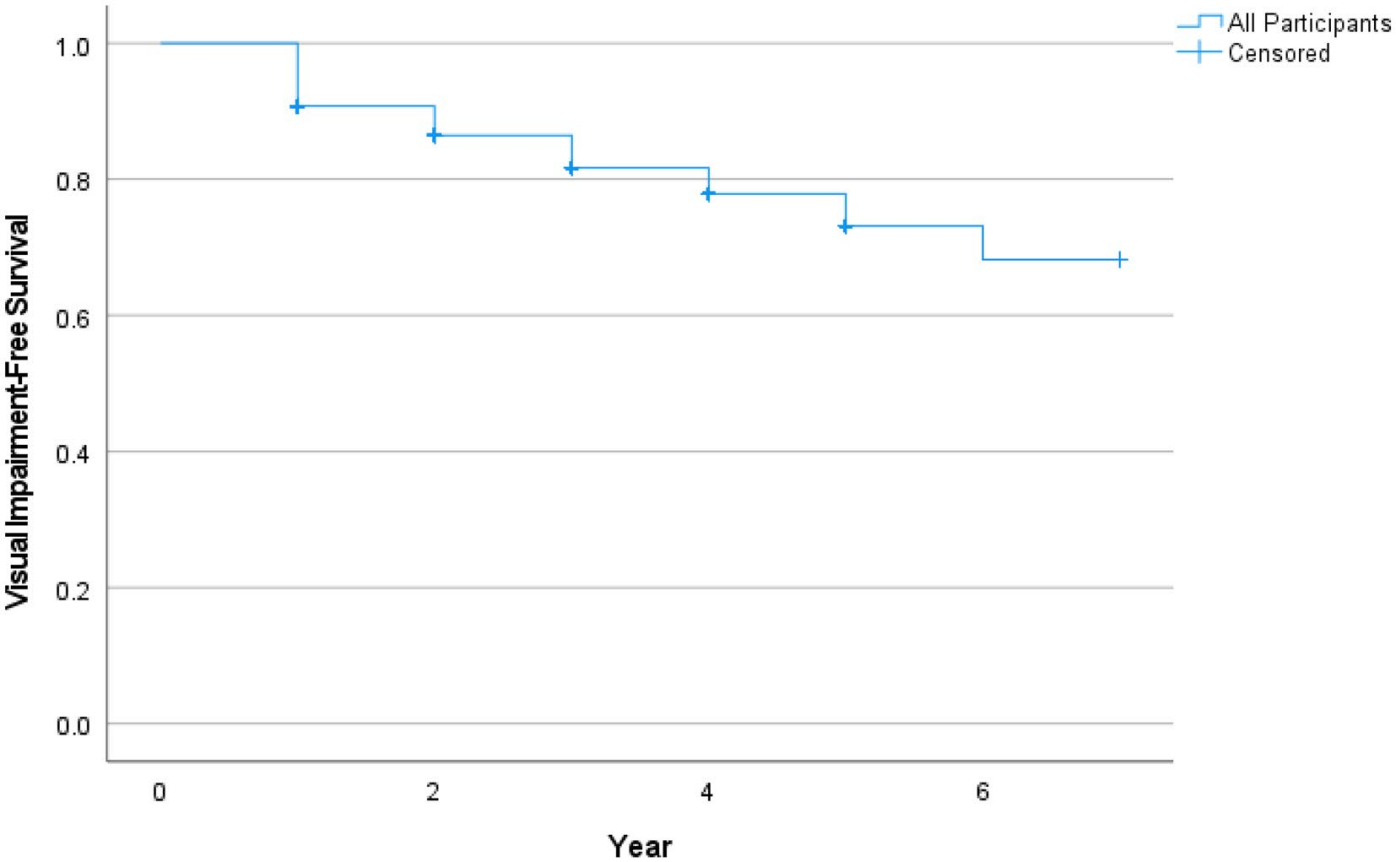
A Kaplan–Meier curve illustrating visual impairment-free survival over 6 years.

Over the 6-year follow-up, 3151 (29.2%) developed visual impairment. Figure 1 showed the Kaplan–Meier curve of visual impairment-free survival over 6 years.

Compared to the group without incident visual impairment, the group that developed visual impairment was older and was predominantly female, with lower educational attainment, poorer visual acuity, higher prevalence of ophthalmological diseases (cataract and glaucoma) and physical impairment, and lower adherence to healthy lifestyle practices (less participation in physical exercise and intellectual activities) at baseline (Table 1). The group with incident visual impairment did not have shorter years of follow-up than the group without (5.0 years [5.0–6.0 years] vs 5.0 years [4.0–6.0 years], respectively).

**Table 1:** Comparison of baseline characteristics between participants with and without incident visual impairment over 6 years.

Baseline characteristics	Total study sample	Incident visual impairment		P value
	N = 10,806	No (*n* = 7655)	Yes (*n* = 3151)	
Dementia, n (%)	194 (1.8)	122 (1.6)	72 (2.3)	0.01
Visual acuity in LogMAR, mean (SD)	0.37 (0.08)	0.36 (0.09)	0.39 (0.05)	< 0.001
Cataract, n (%)	6170 (57.1)	4174 (54.5)	1996 (63.3)	< 0.001
Glaucoma, n (%)	326 (3.0)	213 (2.8)	113 (3.6)	0.03
Age, mean (SD)	73.6 (4.5)	73.3 (4.4)	74.4 (4.5)	< 0.001
Female, n (%)	6556 (60.7)	4545 (59.4)	2011 (63.8)	< 0.001
No schooling received, n (%)	2420 (22.4)	1556 (20.3)	864 (27.4)	< 0.001
On social welfare, n (%)	1246 (11.5)	876 (11.4)	370 (11.7)	0.66
Diabetes mellitus, n (%)	1592 (14.7)	1106 (14.4)	486 (15.4)	0.19
Hypertension, n (%)	6856 (63.4)	4851 (63.4)	2005 (63.6)	0.80
Hypercholesterolemia, n (%)	4579 (42.4)	3255 (42.5)	1324 (42.0)	0.63
Heart diseases, n (%)	1193 (11.0)	863 (11.3)	330 (10.5)	0.23
Stroke, n (%)	322 (3.0)	229 (3.0)	93 (3.0)	0.91
Parkinson’s disease, n (%)	46 (0.4)	34 (0.4)	12 (0.4)	0.65
Depression, n (%)	404 (3.7)	269 (3.5)	135 (4.3)	0.06
Hearing impairment, n (%)	2280 (21.1)	1582 (20.7)	698 (22.2)	0.09
Physical impairment, n (%)	655 (6.1)	431 (5.6)	224 (7.1)	0.003
Physical exercises, n (%)	5412 (53.4)	3905 (54.5)	1507 (50.8)	0.001
Intellectual activities, n (%)	7562 (70.0)	5429 (70.9)	2133 (67.7)	0.001
Social activities, n (%)	8256 (76.4)	5849 (76.4)	2407 (76.4)	0.98
Adequate fruit and vegetable intake, n (%)	5374 (49.7)	3814 (49.8)	1560 (49.5)	0.78
Smoking, n (%)	550 (5.1)	396 (5.2)	154 (4.9)	0.54

### Association of baseline dementia with incident visual impairment

Baseline prevalence of dementia was higher in the group that developed visual impairment than the group that remained free of visual impairment (72 out of 3151 [2.3%] vs 122 out of 7655 [1.6%]; *P* = 0.01; Table 1).

**Figure 2:**
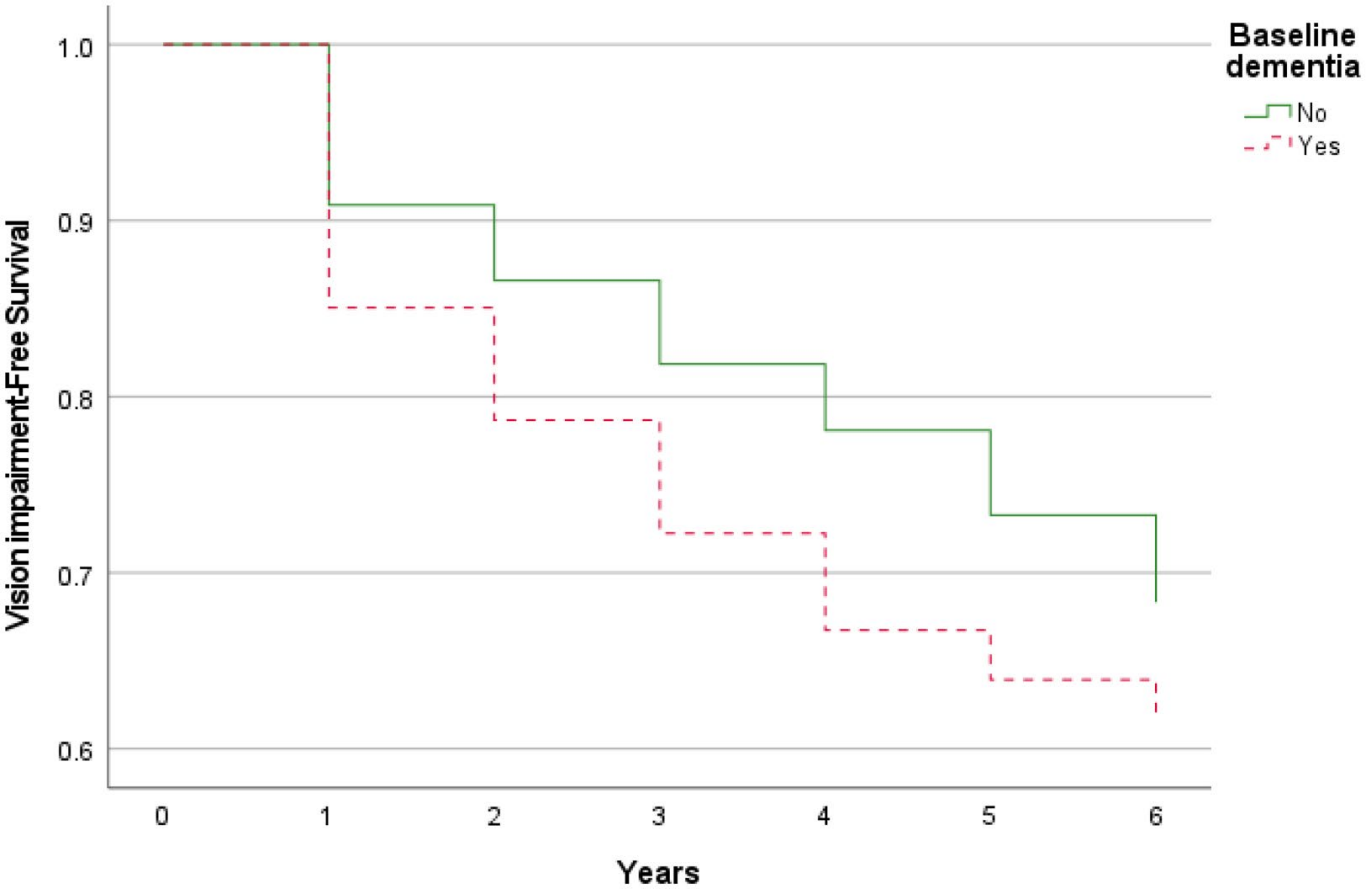
Differences between dementia and dementia-free groups in terms of the distribution of time till visual impairment (the event) occurred.

The unadjusted Hazard Ratio (HR) for incident visual impairment by baseline dementia was 1.33 (95% CI 1.05–1.68; *P* = 0.02). Figure 2 illustrated the differences in distribution of time till the development of visual impairment between participants with and without dementia at baseline.

However, the association between dementia and incident visual impairment was no longer significant at the 5% level after adjusting for baseline visual acuity and additionally for different potential confounding factors in the multivariate analyses (Table 2). The attenuation of HR appears to be driven mainly by baseline visual acuity (HR 3.26; 95% CI 2.78–3.83; *P* < 0.001), and to a lesser degree by presence of cataract (HR 1.29; 95% CI 1.20–1.39; *P* < 0.001), age (HR 1.04; 95% CI 1.03–1.04; *P* < 0.001), sex (female; HR 1.11; 95% CI 1.02–1.21; *P* = 0.02), and low educational level (HR 1.22; 95% CI 1.12–1.34; *P* < 0.001).

**Table 2:** Hazard ratios (HRs) of baseline dementia for incident visual impairment over 6 years.

Dementia	HR	95% CI	P value
Unadjusted	1.33	1.05–1.68	0.02
**Adjusted for**^**†**^			
Baseline visual acuity	1.23	0.97–1.55	0.09
Cataract and glaucoma	1.22	0.97–1.54	0.10
Age, female, educational level, low socioeconomic status	0.98	0.78–1.25	0.90
Diabetes, hypertension, hypercholesterolemia, heart diseases, stroke, Parkinson’s disease, and depression	0.98	0.77–1.25	0.89
Hearing and physical impairments	0.99	0.78–1.25	0.91
Regular participation in physical exercise, intellectual activities and social activities, adequate daily intake of fruits and vegetables, and smoking	0.93	0.72–1.21	0.61

†Models were adjusted for baseline visual acuity and additionally for each of the following groups of covariates.

## Discussion

In this 6-year longitudinal follow-up study of over 10,000 community-living older adults, we did not find evidence of dementia independently increasing the risk of incident visual impairment. This was contrary to our working hypothesis and the prior findings of the Salisbury Eye Evaluation Study and the National Health and Aging Trends Study^12,13^. Our present findings highlight the need for re-evaluating whether dementia is indeed a risk factor for visual impairment.

### Comparison with previous studies

Consistent with the Salisbury Eye Evaluation Study and the National Health and Aging Trends Study^12,13^, we found that older adults experienced worsening of visual acuity over time, as evidenced by a growing proportion of participants developing visual impairment in successive years. However, in contrast to these two studies, we did not find an independent association between dementia and incident visual impairment.

Several reasons might account for the difference in findings between ours and the prior studies. First, unlike the Salisbury Eye Evaluation Study, which examined the association between the MMSE score and visual acuity changes^12^, we investigated the association between dementia and incident visual impairment. Specifically, we performed comprehensive clinical cognitive assessment and identified dementia in accordance with the international diagnostic criteria. These helped to minimize the potential bias of scoring worse in MMSE due to other non-cognitive factors such as age, educational level, and poor vision. Second, unlike the National Health and Aging Trends Study, which assessed visual difficulty by self-reporting^13^, we conducted visual acuity tests and defined moderate visual impairment in accordance with the World Health Organization criteria^9^. As some people with dementia might have anosognosia, apathy, or impaired judgment, they might have difficulty in describing their visual function accurately, thereby introducing self-report bias. Also, previous studies have found that people with dementia are less likely to report, seek, and adhere to treatment due to their underlying cognitive impairments^16^; one study conducted in nursing homes found that a significant proportion of those with dementia use glasses infrequently, improperly, or have prescriptions that are no longer sufficient to correct their vision^17^. The visual difficulty experienced by people with dementia might be explained by failure to obtain or wear glasses rather than a decline in visual acuity. Our standardized clinical assessment of visual acuity was therefore helpful in minimizing the risk of misclassification error in self-reporting. Third, to reduce the possibility of reverse causality, we excluded participants who had already been identified to have visual impairment at baseline and those who developed incident dementia at follow-up. Last, our study was better controlled than the previous studies, with consideration of different lifestyle behaviours and additional health problems, such as depression which is associated with lower treatment adherence^18^. Our study, amid the negative findings, was therefore clinically important and provided additional insight to the question of whether dementia increases the risk of visual impairment longitudinally.

We have previously identified and reported elsewhere that moderate-to-severe, but not mild, visual impairment is independently associated with higher risk of incident dementia in older adults^8^, suggesting that visual impairment is a potentially modifiable risk factor for dementia. While our present study did not find an association the other way around, our findings are in line with those from the Salisbury Eye Evaluation Study, which suggest that the influence of visual impairment on changes in cognitive function is potentially greater than the reverse^12^. Interestingly, among the various confounding variables that we examined in the present study, baseline visual acuity appears to be the key factor that influences the incidence of visual impairment, suggesting that poor prior vision might play a more important role than dementia in predicting risk of visual impairment or contributing to the development of visual impairment. Our findings highlight the potential importance of preventing visual impairment through early correction of poor visual acuity in people living with dementia. Healthcare professionals and caregivers need to be mindful of poor vision in older adults with dementia, and vision screening might be beneficial for this special population in identifying reversible or treatable causes of poor vision and thereby preventing progression to visual impairment.

### Strengths and limitations

This study had several strengths. We followed a large territory-wide community cohort for 6 years, with a low attrition rate. We used a comprehensive and in-depth clinical interview to ascertain the diagnosis of dementia, and we excluded those who developed dementia during the follow-up period. We performed regular standardized assessment on visual acuity rather than relying on self-report to identify visual impairment, and we excluded those who had already had visual impairment at baseline. Moreover, a wide range of health problems, sociodemographic factors, and health behaviours were included in this study.

Nevertheless, this study had some limitations. Firstly, our participants were all ethnic Chinese, living in the community, with a relatively low educational level, and a low prevalence of dementia at baseline. Caution is required when applying our findings to the other populations of older adults. Secondly, the possibilities of misclassification error, reverse directionality, and residual confounding in the dementia-visual impairment association, despite minimized in this study, could not be completely excluded in an observational study. We did not examine the subtype, onset, duration, and treatment of dementia, and we did not have information on the onset, duration, and treatment of poor visual acuity at baseline. Although cataract and glaucoma were included in this study, we did not assess other common causes of visual impairment in older adults (such as age-related macular degeneration, diabetic retinopathy, etc.). And although we included lifestyle behaviours as potential confounders in our analysis, the information was collected based on self-reporting and thus susceptible to biases. Thirdly, visual acuity was measured using the Snellen chart rather than the LogMAR chart, though we converted the Snellen acuity to LogMAR for analysis. Last but not least, based on our previous findings of moderate-to-severe visual impairment being a potential risk factor for incident dementia^8^, we defined visual impairment of at least moderate severity as the outcome of our present study. As the majority of our participants had at least mild visual impairment at baseline (the World Health Organization definition of mild visual impairment was visual acuity of ≥ 0.3 in LogMAR^9^; the mean visual acuity of our cohort was 0.37 in LogMAR), and few participants developed severe visual impairment or complete blindness (≥ 1.0 in LogMAR) in 6 years, future research should examine whether a dementia-visual impairment association exists when alternative definitions of visual impairment are used as the study outcome or when the follow-up time is extended beyond 6 years.

### Conclusions and implications

While we had previously identified an association of visual impairment with higher risk of incident dementia^8^, our present study did not find an association of dementia with higher risk of incident visual impairment. Taken together, our findings suggest that further proof is needed before one can conclude that dementia is a risk factor for visual impairment or a bi-directional association exists between dementia and visual impairment in older adults.

## Methods

### Study design, setting and sample

This 6-year longitudinal observational study was based on the territory-wide community cohort of the Elderly Health Centres (EHCs) of the Department of Health of the Government of Hong Kong, which has been reported elsewhere^8^. Briefly, our cohort consisted of 18,298 individuals aged 65 years or older from all 18 districts in Hong Kong at inception. All participants completed standardized clinical assessment of cognitive status and comprehensive evaluation of a wide range of physical and mental health problems, lifestyle behaviours, and sociodemographic factors at the EHCs at baseline. They have since then been followed yearly at the EHCs. In this study, those who missed follow-up were actively traced and interviewed, and written informed consent was obtained from all the participants of the study, or from the Legally Authorized Representatives if they were mentally incapable of giving consent, before interview. The names of those not traceable were verified with the Deaths Registry for the cause of death.

The study was approved by the Ethics Committee of the Institutional Review Board of the Government and the University. The study was conducted in accordance with the tenets of the Declaration of Helsinki.

Since this study aimed to examine the association between baseline dementia and incident visual impairment in 6 years in Chinese, we excluded participants who were not ethnic Chinese (n = 60), had visual impairment at baseline (n = 6367), and developed incident dementia at follow-up (n = 1065). Hence, a total of 10,806 participants without visual impairment at baseline were included.

### Identification of visual impairment

Visual acuity was assessed yearly for both eyes using the line scores from the 6-m Snellen E chart mounted on a wall at the EHCs. Given the non-normal distribution, the Snellen fractions were converted to LogMAR, with a higher score indicating poorer visual acuity. In this study, we defined visual impairment as the better eye, despite best correction, having a visual acuity of equal to or worse than 6/18 (or LogMAR ≥ 0.5), which was equivalent to at least moderate visual impairment according to the World Health Organization (WHO) definition^9^. The study outcome was incident visual impairment over 6 years.

### Identification of dementia

To reduce the potential risk of misclassifying participants with poor vision as having dementia due to poorer performance in vision-dependent cognitive tasks, we performed a comprehensive clinical examination, which included detailed history taking to look for evidence of impaired functioning owing to significant memory decline or executive dysfunction rather than sensory impairments, and nonvisual cognitive testing, such as the Delayed Word Recall Test and the Abbreviated Mental Test, at the EHCs at baseline. The same examination was repeated at the EHCs at follow-up, as participants who were found to have developed incident dementia would be excluded in the data analysis as a measure to minimize the risk of reverse causation. Participants who missed this but agreed to a follow-up interview at the EHCs or at home upon tracing received a clinical examination and were administered the Clinical Dementia Rating by geriatric psychiatrists. Dementia was diagnosed according to the International Statistical Classification of Diseases and Related Health Problems, Tenth Revision (ICD-10) and a Clinical Dementia Rating (CDR) of 1 to 3^19^. A panel of geriatric psychiatrists reviewed all the diagnosis of dementia independently. For cases whose diagnosis was uncertain or in disagreement, the principal investigator adjudicated the final diagnosis.

### Assessment of other variables

Information on sociodemographic factors, clinical risk factors, hearing and physical impairments, and lifestyle behaviours was obtained during assessments. Demographic factors included age, sex, and educational level. Low socioeconomic status was defined as receiving Comprehensive Social Security Assistance (CSSA) from the government. Clinical risk factors included cataract, glaucoma, diabetes, hypertension, hypercholesterolemia, heart diseases, stroke, Parkinson’s disease, and depression; they were diagnosed with reference to the ICD-10 criteria by ophthalmologists, physicians and psychiatrists accordingly. Hearing impairment was defined as 1- and 2-kHz loss of more than 40 decibels in the better ear during audiometric testing (Audioscope, Welch Allyn 23300). Physical impairment was defined as needing an aid to walk or being chairbound. A standardized self-reported questionnaire was used to assess the participants’ lifestyle behaviours in the prior month. The classification system for different lifestyle behaviours has already been validated for Hong Kong Chinese older people^20^. The criteria for regular participation in physical, intellectual and social activities, adequate amount of fruit and vegetable intake, and smoking were defined as previously reported^21–23^.

### Statistical analyses

Sample size estimation was performed using the G*Power software. Sample size was calculated based on estimates of annual decline in visual acuity (~ 0.01 LogMAR per year) and its correlation with MMSE changes from a previous longitudinal study^12^ and the point prevalence of dementia and the mean and standard deviation of visual acuity in our participants at baseline from our previous study^8^. As older adults with dementia have lower MMSE score with greater rate of decline than those without, we estimated that the proportion of participants with incident visual impairment in 6 years would be approximately 50% greater in the dementia group than the dementia-free group. With alpha set at 0.05, a sample of 4000 participants would yield at least 80% power for detecting a significant difference in 6-year incidence of visual impairment between those with and without dementia at baseline.

Statistical analysis was performed using IBM SPSS Statistics, Version 26.0 (IBM Corp). Incidence of visual impairment (i.e., the event) was computed based on the number of participants who developed incident visual impairment over 6 years. A Kaplan–Meier curve was constructed to illustrate the visual impairment-free survival over 6 years. Data was censored when the participant remained free of visual impairment by the time the study ended, defaulted follow-up, or died without visual impairment before the study ended.

Baseline dementia and other variables were compared between participants with and without incident visual impairment using either the independent *t*-test or the *X*^2^ test, as appropriate. The level of statistical significance was set at *P* < 0.05 (2-tailed).

Cox regression analysis was performed to examine the influence of dementia (and other variables) at baseline on time to visual impairment. In our analysis, our primary outcome was incident visual impairment, and the predictor variable was presence of dementia at baseline. The hazard ratios (HRs) were computed to yield point estimates with 95% confidence intervals (95% CIs), and the differences in visual impairment-free survival over 6 years in relation to presence of baseline dementia were plotted. To control for the potential confounding effects, we conducted the multivariate analyses and calculated the adjusted HRs by including baseline visual acuity and each of the covariates (cataract, glaucoma, diabetes, hypertension, hypercholesterolemia, heart diseases, stroke, Parkinson’s disease, depression, hearing impairment, physical impairment, participation in physical exercises, intellectual and social activities, adequate daily intake of fruits and vegetables, smoking, age, female, educational level, and low socioeconomic status) into the model.

### Ethical approval

This study was approved by the Ethics Committee of the Department of Health of the Government of Hong Kong (L/M 623/2010) and the Joint Clinical Research Ethics Committee of the Chinese University of Hong Kong and the New Territories East Cluster of the Hospital Authority (CRE-2011.036).

## Data Availability

The data that support the findings of this study are available from the corresponding author upon reasonable request.
